# Supplementary material for: Carbapenem-resistant enterobacteriaceae: analyzing knowledge and practice in healthcare providers
Source: PeerJ. 2014 May 22;2:e405. doi: 10.7717/peerj.405 (PMC4034608; doi:10.7717/peerj.405)
Supplement: Supplemental Information [file peerj-02-405-s001.doc]

Antibiotic resistance is increasing worldwide. Much focus has been on gram-positive organism resistance. We are interested in the recently recognized rise in gram-negative organism resistance and therefore are conducting a survey of all physicians, nurse practitioners, and physician assistants regarding their knowledge of and practice regarding such organisms at Tufts Medical Center and four other Tufts-associated hospitals. While participation in this survey is voluntary, your responses will be used to research, prevent, and treat resistant organisms. The survey consists of 20-25 questions, depending on your responses, and will take you between 10-12 minutes to complete.

In addition, by providing your email in a separate link, so to keep your responses anonymous, your name will be entered into a raffle to win one of three $150 Amazon gift card

Please email any questions or comments to Evangeline Thibodeau at [Ethibodeau@tuftsmedicalcenter.org](mailto:Ethibodeau@tuftsmedicalcenter.org)

Thank you for your time and participation in this project.

1) What is your professional title?

a) MD

b) DO

c) NP

d) PA

e) other: please specify

2) What year did you finish your education? For example, when did you graduate medical school or finish your NP/PA training? _____________

3) Since completing your education, how many years have you been practicing your current specialty? _________________

4) What department do you currently work in?

a) Internal Medicine

b) Internal Medicine subspecialty: please specify _____________­­­­­­­­­­­

c) Surgery

d) Surgical subspecialty: please specify ___________

e) Pediatrics

f) Pediatrics subspecialty: please specify____________

g) Psychiatry

h) Obstetrics/Gynecology

i) Neurology

j) Other: please specify __________

5) Since finishing your education, have you worked in other specialties other than your current department?

a) No, I have only practiced in my current department

b) Yes

6) If yes to question 5, what other specialties have you previously worked in? **(check all that apply)**

a) Internal Medicine

b) Internal Medicine subspecialty: please specify _____________­­­­­­­­­­­

c) Surgery

d) Surgical subspecialty: please specify ___________

e) Pediatrics

f) Pediatrics subspecialty: please specify____________

g) Psychiatry

h) Obstetrics/Gynecology

i) Neurology

j) Other: please specify __________

The following questions are to assess your knowledge and practice regarding treatment of resistant gram-negative organisms. We expect that various areas of expertise and level of training will affect your knowledge of and experience with treating such infections. However to better understand the gaps in knowledge and where to focus our teaching efforts, we would like you to answer the following questions as honestly as possible. The results of this survey in no way will be graded or identified. They will be kept confidential and only a summary of the results will be reported in a scientific nature.

7) What is an extended spectrum beta-lactamase (ESBL)-producing organism?

a) An organism that is resistant to Vancomycin

b) An organism that carries an enzyme conferring resistance to most beta-lactam antibiotics including penicillins, cephalosporins, and monobactams such as aztreonam

c) An organism that produces an enzyme conferring resistance to all quinolone antibiotics.

d) An organism that is resistant to all cephalosporins except the 4^th^ generation cephalosporins such as cefipime but is sensitive other beta-lactam antibiotics such as monobactams (i.e. aztreonam)

e) An organism that produces an enzyme that makes it is sensitive only to Daptomycin

f) Do not know

8) What are Carbapenem Resistant Enterobacteriaceae (CRE)?

a) Organisms that produces an enzyme conferring resistance to both Vancomycin and Daptomycin

b) Organisms that produce an enzyme conferring resistance to all cephalosporins and all quinolones

c) Organisms that produces an enzyme conferring resistance to most B-lactam antibiotics including penicillins, cephalosporins, and monobactams such as aztreonam.

d) Organisms that produces an enzyme conferring resistance to all ß-lactam molecules including carbapenems such as Meropenem and Ertapenem.

e) Do not know

9) What is an example of a CRE?

a) New Delhi Metallo-protease (NDM)

b) Athens betalactamase (ABL)

c) Klebsiella producing carbapenamase (KPC)

d) b and c

e) a and c

f) all of the above

g) Do not know

10) If your patient was infected with an ESBL, what antibiotic or class of antibiotics would be most appropriate therapy? (check all that apply)

a) 4^th^ Generation cephalosporins such as cefipime

b) Quinolones

c) Tigecycline

d) Daptomycin

e) Carbapenems such as Meropenem or Ertapenem

f) Colistin

g) Do not know

11) If your patient was infected with a CRE, what antibiotic or class of antibiotics would be most appropriate therapy? (check all that apply)

a) 4^th^ Generation cephalosporins such as cefipime

b) Quinolones

c) Tigecycline

c) Daptomycin

e) Carbapenems such as Meropenem or Ertapenem

f) Colistin

g) Do not know

12) Which of the following statements best describes your opinion regarding gram negative resistance?

a) I didn’t know there was gram-negative resistance until I took this survey

b) I am not concerned about gram negative resistance

c) I am a little concerned about gram-negative resistance
  d) I am moderately concerned about gram-negative resistance
  e) I am very concerned about gram-negative resistance

13) How strongly do you agree with the following statement: “There are sufficient new antibiotics in development to treat resistant gram negative infections:

a) I do not agree with this statement at all

b) I agree with this statement a little

c) I agree with this statement a lot

d) I agree with this statement completely

e) I have no idea

14) Prior to prescribing or ordering any antibiotic how often do you consider whether your patient is at risk for a resistant organism?

a) never

b) sometimes but less than half of the time

c) about half of the time

d) more often than not

e) always

f) I have never prescribed or ordered an antibiotic

15) If yes to 14 (answer b-e), how do you determine if they are at risk? (**check all that apply)**

a) Review prior microbiological reports

b) Review prior antibiotic exposures

c) Review prior discharge summaries and outpatient notes

d) Consider recent contact with the healthcare environment

e) Other: please explain

16) What patients or populations do you consider at risk for resistant organisms? **(check all that apply)**

a) Patients on hemodialysis

b) Patients who reside in a nursing home

c) Patients who have received antibiotics in the past year

d) Patients who have been admitted to the hospital within 30 days

e) Community-residing patients who have not received antibiotics within 90 days

f) Immunocompromised patients

g) Patients in the ICU

h) None of the above

i) All of the above

17) Prior to prescribing an antibiotic for your patient how often do you review their antimicrobial history that does not pertain to the current infection?

a) never

b) sometimes but less than half of the time

c) about half of the time

d) more often than not

e) always

18) When you do not always review the antimicrobial history (answer a-d to 17), what are the reasons? **(check all that apply)**

a) I don’t know how to interpret them

b) Not enough time

c) I always call a specialist to help me when I need it

d) When the records are not available

e) I do not consider them relevant

f) Other: please explain________

19) Prior to prescribing an antibiotic for your patient how often do you review the antimicrobial history for the current infection?

a) never

b) sometimes but less than half of the time

c) about half of the time

d) more often than not

e) always

20) When you do not always review the current record (answer a-d to 19), what are the reasons? **(check all that apply)**

a) I don’t know how to interpret them

b) Not enough time

c) I always call a specialist to help me

d) When the records are not available

e) I do not consider them relevant

e) Other: please explain________

21) When you review the microbiological records, which of the following methods do you use most often?

a) I never review the microbiological reports

b) I look at the drugs that are reported as sensitive and use those

c) I look at the MIC cutoffs to determine which drugs are sensitive

d) I look at the MIC cutoffs of each drug and see if it is appropriate for the bacteria I want to treat

e) I call the micro lab to discuss the data

f) Other: please explain

22) If you have started empiric antibiotics, prior to knowing results of the culture data, what best describes your practice of deescalating or targeting antibiotics when the culture and susceptibility report is later available?

a) I never deescalate or target antibiotics

b) I call for help from a specialist such as an ID consult or the Antimicrobial Management Team (AMT) the majority of the time

c) I feel fairly comfortable reviewing the report and changing antibiotics myself the majority of the time

d) Other: Please explain

23) When prescribing an antibiotic for any reason, how often do you call a specialist such as an ID consult or AMT?

a) never

b) sometimes but less than half of the time

c) about half of the time

d) more often than not

e) always

24) What is the main reason you call an ID or AMT consult when prescribing antibiotics (excluding the requirement for antimicrobial approval)?

_________________________________________

Separate link for the following question:

25) Please provide your email address if you wish to enter into the raffle:­­­­­­­­­___________
